# *EARLY FLOWERING 3* and Photoperiod Sensing in *Brachypodium distachyon*

**DOI:** 10.3389/fpls.2021.769194

**Published:** 2022-01-06

**Authors:** Frédéric Bouché, Daniel P. Woods, Julie Linden, Weiya Li, Kevin S. Mayer, Richard M. Amasino, Claire Périlleux

**Affiliations:** ^1^Laboratory of Plant Physiology, InBioS-PhytoSYSTEMS, Department of Life Sciences, University of Liège, Liège, Belgium; ^2^Plant Sciences Department, University of California, Davis, Davis, CA, United States; ^3^Laboratory of Genetics, University of Wisconsin, Madison, WI, United States; ^4^Department of Biochemistry, University of Wisconsin, Madison, WI, United States; ^5^United States Department of Energy Great Lakes Bioenergy Research Center, University of Wisconsin, Madison, WI, United States; ^6^Howard Hughes Medical Institute, Chevy Chase, MD, United States

**Keywords:** *Brachypodium*, flowering, circadian clock, photoperiod, *ELF3*, Pooideae, temperate grasses

## Abstract

The proper timing of flowering, which is key to maximize reproductive success and yield, relies in many plant species on the coordination between environmental cues and endogenous developmental programs. The perception of changes in day length is one of the most reliable cues of seasonal change, and this involves the interplay between the sensing of light signals and the circadian clock. Here, we describe a *Brachypodium distachyon* mutant allele of the evening complex protein EARLY FLOWERING 3 (ELF3). We show that the *elf3* mutant flowers more rapidly than wild type plants in short days as well as under longer photoperiods but, in very long (20 h) days, flowering is equally rapid in *elf3* and wild type. Furthermore, flowering in the *elf3* mutant is still sensitive to vernalization, but not to ambient temperature changes. Molecular analyses revealed that the expression of a short-day marker gene is suppressed in *elf3* grown in short days, and the expression patterns of clock genes and flowering time regulators are altered. We also explored the mechanisms of photoperiodic perception in temperate grasses by exposing *B. distachyon* plants grown under a 12 h photoperiod to a daily night break consisting of a mixture of red and far-red light. We showed that 2 h breaks are sufficient to accelerate flowering in *B. distachyon* under non-inductive photoperiods and that this acceleration of flowering is mediated by red light. Finally, we discuss advances and perspectives for research on the perception of photoperiod in temperate grasses.

## Introduction

In many flowering plant species, photoperiod sensing is key to the synchronization of reproduction with seasonal changes in order to maximize reproductive success. Sensitivity to photoperiod has long been a major agricultural trait selected by breeders to improve yields or adapt crop varieties to different latitudes (e.g., Turner et al., 2005; Lundqvist, 2009; Wilhelm et al., 2009; Casao et al., 2011; Faure et al., 2012). In Pooideae, a monophyletic group of temperate grasses that includes the model grass *Brachypodium distachyon* (*B. distachyon*) as well as important cereal crops such as wheat, oat, and barley, the lengthening of photoperiod in the spring is a signal that stimulates flowering, so that seeds are produced and ripen under favorable conditions (e.g., Ream et al., 2012). Although the transduction mechanisms and pathways through which the perception of day length regulates developmental processes remain relatively poorly understood in temperate grasses, this has been the focus of extensive research in other groups of plants, especially in the model Brassicaceae *Arabidopsis thaliana* (Arabidopsis; Song et al., 2015).

Light signals, which are perceived by photoreceptors, are integrated into circadian clock-regulated processes to be translated into developmental responses (Paik and Huq, 2019). The perception of photoperiod and light quality are achieved through complementary photoreceptors: phytochromes are responsible for the perception of red/far-red wavelengths while cryptochromes, phototropins, and ZEITLUPEs (ZTLs) mediate responses to blue light (Quail, 2002; Möglich et al., 2010). The phytochromes are photolabile photoreceptors existing in two reversible states: the active Pfr form, which is formed under red light, and the inactive Pr form, which accumulates under far-red light or through the temperature-mediated reversion of the Pfr form (Cheng et al., 2021). In Arabidopsis, five phytochromes (PhyA-E) contribute to the modulation of important developmental processes, such as photomorphogenesis, gravitropism, circadian clock entrainment, and flowering time regulation (Legris et al., 2019). Phytochromes form dimers which, upon activation by red light, can be translocated toward the nucleus where they modulate gene expression through their interaction with protein partners such as PHYTOCHROME INTERACTING PROTEINS (PIFs) (Leivar and Quail, 2011; Cheng et al., 2021). PIFs are basic helix-loop-helix transcription factors that typically act as regulators of light responses by direct binding to the promoter of target genes. PIFs are degraded upon interaction with light-activated phytochromes, leading both to broad transcriptional reprogramming (Lucas and Prat, 2014) and to modifications of the chromatin landscape (Willige et al., 2021). Although five phytochromes were identified in Arabidopsis, only *PHYA*, *PHYB*, and *PHYC* are conserved in temperate grasses (Mathews, 2010), among which *PHYB* and *PHYC* were shown to be key to control flowering time under long days (LD; Chen et al., 2014; Woods et al., 2014; Pearce et al., 2016; Kippes et al., 2020).

Photoreceptor signaling provides input into the biological clock that controls circadian rhythms, which enables organisms to anticipate daily changes in the environment and thus avoid possible stresses (Johansson and Staiger, 2014). There is an interdependent regulatory loop between photoreceptors and the clock, photoreceptors reporting the changes in the length of days that enable the clock to adapt to seasonal changes (Oakenfull and Davis, 2017). Indeed, in Arabidopsis, while the clock is reset every morning by light, the induction of *PHYA* expression at night leads to the accumulation of phyA protein in the morning, so that the pool of activated phytochrome at dawn is sufficient to trigger morning genes (Seaton et al., 2018). The daily oscillation of the clock is controlled through a complex array of interactions, which is often summarized as three interlocked feedback loops (Creux and Harmer, 2019). The morning-expressed *CIRCADIAN CLOCK ASSOCIATED1* (*CCA1*) and *LATE ELONGATED HYPOCOTYL* (*LHY*) genes encode inhibitors of the *PSEUDO-RESPONSE REGULATORs* (*PRRs*) *PRR7* and *PRR9*, which themselves repress *CCA1* and *LHY*, thus forming the morning loop. The central oscillator is formed by mutual repression between *CCA1/LHY* and *TIMING OF CAB EXPRESSION1* (*TOC1*). TOC1 also inhibits the expression of *GIGANTEA* (*GI*) and the components of the evening complex (EC), *LUX ARRHYTHMO* (*LUX*), *EARLY FLOWERING3* (*ELF3*), and *ELF4*, whose expression peaks at dusk (Huang and Nusinow, 2016). The loss of any of these components impairs the function of the EC (Hicks et al., 1996; Covington, 2001; Doyle et al., 2002; Hazen et al., 2005) and thus causes circadian clock malfunction by preventing the EC-mediated repression of *PRR7*/*9*, *GI*, *TOC1*, and *LUX* (Huang and Nusinow, 2016). These intricate interactions produce daily rhythms that are synchronized with changes in the photoperiod and/or temperature to control the expression of thousands of genes (Covington et al., 2008).

The role of circadian rhythms in the photoperiodic induction of flowering has been most extensively studied in Arabidopsis. For example, the circadian clock entrains the expression of *CONSTANS* (*CO*), whose transcripts accumulate at dusk but whose protein is only stable in the light (Suárez-López et al., 2001; Valverde et al., 2004). Night-break experiments demonstrated that providing a short period of light at a specific time of the night was sufficient to induce flowering (Goto et al., 1991) and suggested that a process known as external coincidence (Bunning, 1937) is operating in Arabidopsis. It was later demonstrated that the coincidence between light and sufficient CO protein levels, which typically occurs in nature under the extended photoperiod of the spring, leads to the stabilization of CO that activates the expression of *FLOWERING LOCUS T* (*FT*) in leaves (An et al., 2004). *FT* encodes a protein annotated as a phosphatidylethanolamine-binding protein and now referred to as florigen, which is transported from leaves to the shoot apical meristem to induce the floral transition (Corbesier et al., 2007; Tamaki et al., 2007).

In long-day flowering temperate grasses, the photoperiod-mediated floral transition starts with the perception of light signals by phyB and phyC, which can form heterodimers (Nishida et al., 2013; Chen et al., 2014; Woods et al., 2014; Kippes et al., 2020). Active alleles of these two phytochromes are required for the induction of the pseudo-response regulator *PHOTOPERIOD1* (*PPD1*) under LD (Chen et al., 2014; Pearce et al., 2016). Once induced by LD, PPD1 stimulates the expression of the florigen *FT1*, but whether or not this induction is direct is not known. FT1 then interacts with the transcription factor FD and, together, they trigger the expression of the MADS-box protein encoding gene *VERNALIZATION1* (*VRN1*) in the leaves (Li and Dubcovsky, 2008). VRN1 in turn upregulates the expression of *FT1* in a positive feedback loop that eventually overcomes the repressive effect mediated by the zinc-finger-CCT domain transcription factor VRN2 on *FT1* expression (Distelfeld and Dubcovsky, 2010; Ream et al., 2014). The FT1 protein is then thought to migrate to the shoot apical meristem, as shown in Arabidopsis, to induce the expression of *VRN1*, thus promoting flowering under favorable photoperiods (Woods and Amasino, 2015).

The absolute requirement for inductive photoperiods for flowering in *B. distachyon* suggests that this process is tightly controlled by circadian clock mechanisms (Woods and Amasino, 2015). The EC component *ELF3* is a key regulator at the intersection of photoperiod-induced flowering and the circadian clock, and, not surprisingly, this gene has been an important breeding target for crop improvement (Bendix et al., 2015). Indeed, in LD flowering plants, natural variation in *ELF3* allowed growing seed crops in new environments, whether at latitudes where shorter photoperiods would have otherwise prevented flowering, or under stressful conditions in which early flowering represents an advantage (Bendix et al., 2015). For instance, the wild relatives of cultivated peas from temperate regions are obligate LD plants whose domestication as spring crops was associated with natural variation at two photoperiod-sensitivity loci, *HIGH RESPONSE* and *PHOTOPERIOD*, which correspond to two distinct orthologs of *ELF3* (Weller et al., 2012; Rubenach et al., 2017). Natural variation in *ELF3* also allowed adaptation of short-day flowering crops to new cultivation conditions. For instance, in soybean, which is mostly cultivated in temperate climates, natural variation at *ELF3* delayed flowering under the shorter photoperiod of tropical regions, thus enabling an extended flowering phase and increased yields (Lu et al., 2017; Bu et al., 2021). In Arabidopsis, independently of its role as a component of the EC, ELF3 is also able to interact with PIF4 in order to prevent the activation of its transcriptional targets (Nieto et al., 2015). In addition to its role in mediating the photoperiodic response, ELF3 acts as a thermosensor mediating the interplay between the circadian clock, flowering, and ambient temperature (Bullrich et al., 2002; Strasser et al., 2009; Thines and Harmon, 2010; Jung et al., 2020). The Arabidopsis ELF3 protein contains a prion-like domain that, at higher temperatures, undergoes conformational changes that reversibly inactivate ELF3. However, the extent to which this mechanism is conserved across land plants remains to be established as, for instance, the prion-like domain conferring the ambient temperature sensitivity is absent from the *B. distachyon* ELF3 protein (Jung et al., 2020).

Comparative genomics led to the identification of orthologs of circadian clock genes, including *ELF3*, among Arabidopsis, rice, and *B. distachyon* (Higgins et al., 2010), so it is not surprising that *ELF3* is also a key photoperiod response regulator in monocots. In rice, studies on the natural variation of flowering time between Japanese cultivars identified a single-nucleotide polymorphism at the *ELF3* locus as a likely candidate (Matsubara et al., 2012). Indeed, a polymorphism that delayed flowering under short-day inductive photoperiods was caused by a change in *OsELF3* that impedes its ability to control phytochrome-mediated signaling pathways (Saito et al., 2012; Zhao et al., 2012; Itoh et al., 2018). Likewise, certain rapidly flowering barley mutants that were adapted to shorter growing seasons (Gustafsson et al., 1960) were shown to be mutated at the *ELF3* locus (Faure et al., 2012; Zakhrabekova et al., 2012; Boden et al., 2014; Wang et al., 2016). The early flowering phenotype of the barley *elf3* mutant is suppressed by the inhibition of gibberellin biosynthesis, suggesting that the contribution of this hormone is key to the early flowering phenotype caused by the disruption of the clock rhythmicity (Boden et al., 2014). In wheat, the thermosensitive *earliness per se* locus *Eps-A^m^1* (Bullrich et al., 2002) was shown to be linked to mutations in *ELF3* (Alvarez et al., 2016). The conserved role of *ELF3* across the monocot/eudicot divide is indicated by the ability of the *B. distachyon ELF3* gene to rescue the hypocotyl elongation, clock arrhythmicity, and flowering phenotypes of the Arabidopsis *elf3* mutant (Huang et al., 2017). Here, we describe a new mutant allele of *elf3* that was identified in a mutagenized population grown under short days (SD), highlighting that the role of ELF3 in circadian clock function and mediating the photoperiodic induction of flowering genes is conserved.

## Materials and Methods

### Plant Material, Growth Conditions, and Phenotyping

Experiments were conducted using the Bd21-3 accession of *B. distachyon*. An EMS-mutagenized M2 population used for screening was generated as described in Woods et al. (2014). All experiments were carried out using the *elf3* mutant that had been back-crossed twice. For phenotyping and RT-qPCR experiments, plants were grown in 0.5 l pots containing a 4:1 mixture of soil (Brill, Germany) and perlite supplemented with 8 g l^–1^ of Osmocote Exact Standard 5–6 M (ICL Specialty Fertilizers, Israel). Seeds were stratified for 2 days in the dark at 4°C before sowing, and plants were grown under 8, 12, 16, or 20 h photoperiods provided by fluorescent tubes (Philips Master TL-D Super 80 58W 4100K) at an intensity of 150 μmol.m^–2^.s^–1^ (PAR), 70% humidity, 20°C day/night. For the mean internode lengths, we dissected 10–16 plants per genotype at a developmental stage 1–2 leaves before the estimated stage when the *elf3* mutant would transition to flowering based on preliminary experiments (i.e., dissection was performed 43 days after germination under 8 h, 30 days under 12 h, 21 days under 16 h, and 20 days under 20 h), and measured the distance between each node on the main stem. Estimates of chlorophyll contents were performed on the third emerged leaf at the fourth leaf stage using a MC-100 probe (Apogee Instruments, United States).

### Temperature and Night Break Experiments

For vernalization treatments, seeds were stratified for 2 days at 4°C, then placed in soil and cold treated for 3 weeks at 4°C in the dark before transfer to standard growth conditions (150 μmol.m^–2^.s^–1^ light, 70% humidity, 20°C day/night, 8 or 16 h photoperiod). For ambient temperature experiments, Bd21-3 seeds were stratified for 2 days at 4°C and planted in a 16 h photoperiod, 20°C day/night conditions. After 2 weeks, seedlings were transferred to growth chambers at 15, 20, or 25°C day/night. For night-break (NB) experiments performed using fluorescent white light (Philips Master TL-D Super 80 58W 4100K), plants were germinated for 1 week under 10 h SD before being transferred to either 10 h SD, 10 h SD supplemented with a 2 h NB, or 8 h SD supplemented with a 2 h NB. All NBs started at Zeitgeber time (ZT) 16 h, since this time was reported to be the most efficient in other temperate grasses (Pearce et al., 2017). For NB experiments using a red:far-red mixture, plants were grown in 3 l pots under a 12 h photoperiod for 8 weeks. They were subsequently transferred to a 12 h photoperiod supplemented with a 2 h NB given in phytotronic cabinets equipped with Lumiatec PHS::16 (300 W) modular LED luminaries (GDTech, Belgium). NBs were provided at ZT16h using a red to far-red gradient and low light intensities (20 –25 μmol.m^–2^.s^–1^). The spectral distributions of lights (white, red, far-red) provided by LED luminaries are in Supplementary Figure 1A. Controls were either kept under 12 h SD without NB or exposed to a 12 h photoperiod supplemented with a 2 h NB at ZT16h provided by fluorescent tubes (150 μmol.m^–2^.s^–1^). For end-of-day far-red (FR) treatments, plants were grown for 1 week under white light with a 12-h photoperiod before transfer to either 18 h LD or 18 h LD followed by 1-h of FR (Supplementary Figure 1B). All experiments were stopped 100 days after transfer.

### Mapping the *elf3* Mutation and RT-qPCR Analyses

To map the mutation, an M3 homozygous mutant line was crossed with the Bd3-1 accession. The mapping was performed using PCR-amplified InDel markers (Woods et al., 2014) on 40 plants segregating for early flowering under 8 h SD. The candidate genes in this interval were identified using Phytozome (Goodstein et al., 2012), and the coding region of the most likely candidate, *ELF3*, was analyzed by Sanger sequencing. For RT-qPCR, the third leaf at the three-leaf stage of WT and *elf3* plants were harvested every 2 h and pooled separately (*n* = 6–8). RNA was extracted using the NucleoSpin RNA plant kit (Macherey-Nigel, Germany) and reverse transcription was carried out on 1.5 μg of RNA using MMLV RT (Promega, United States), following the manufacturer’s instructions. The RT-qPCR was performed with Takyon Low Rox MasterMix (Eurogentec, Belgium) using 40 cycles of amplification: 10″ at 95°C for denaturation, 20″ at 57°C for hybridization, and 30″ at 72°C for elongation. The geometric mean of *ACT3* and *UBC18* was used to normalize data (Vandesompele et al., 2002). Primers are listed in Supplementary Table 1.

### Generation of *UBI: EARLY FLOWERING 3* Transgenic *elf3* Plants

The *ELF3* coding region cloned into the pENTR/D-TOPO vector, originally published in Huang et al. (2017), was obtained from Dmitri Nusinow. Cloning of *ELF3* into pANIC10a was done as described in Ream et al. (2014). Clones were verified by sequencing and then transformed into chemically competent *Agrobacterium tumefaciens* strain Agl-1. Plant callus transformation of *elf3* with the pANIC10a vector containing the wild type *ELF3* gene was performed as described in Vogel and Hill (2008) by the Great Lakes Bioenergy Research Center for *Brachypodium* transformation facility. Independent transgenic lines were genotyped for the transgene using a gene-specific *ELF3* forward primer and a pANIC10a specific reverse primer (Supplementary Table 1).

## Results

### Identification of the *elf3* Mutation

**FIGURE 1:**
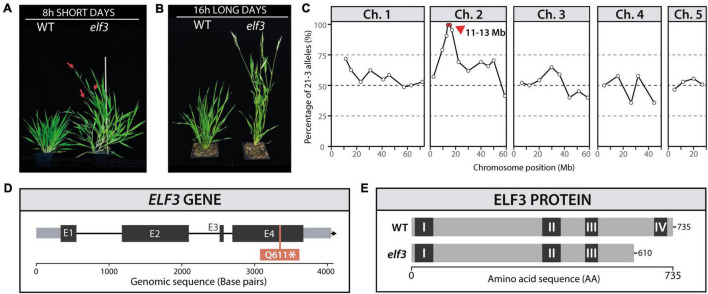
Identification of the *elf3* mutant. Phenotype of the *elf3* mutant and wild-type (WT) *Brachypodium distachyon* plants under **(A)** 8 h (80 days) and **(B)** 16 h photoperiods (50 days). The red arrows indicate early emerging spikes. **(C)** InDel mapping of the segregation of the early flowering phenotype in an M2 mapping population (Bd21-3 and Bd3-1). **(D,E)** Schematic representation of the *ELF3* gene (**D**; E1 to E4 indicate exons) and the ELF3 protein (**E**; regions I to IV indicate conserved protein domains).

*B. distachyon* is an obligate LD species, requiring photoperiods of 14 h or more to flower rapidly (Ream et al., 2014; Woods et al., 2017). In order to further understand the mechanisms controlling this requirement, we screened M2 EMS-mutagenized lines for flowering phenotypes under 8 h SD and identified one rapidly flowering mutant (Figure 1A). To test whether the rapid flowering phenotype was specific to SD, we grew the homozygous mutant lines under a 16 h photoperiod and found that the mutant was also rapid flowering in LD compared to WT plants (Figure 1B). We crossed M3 homozygous mutant lines with the Bd3-1 accession in order to obtain a mapping population. The 1:3 segregation of the rapid flowering phenotype in the F2 population indicated a recessive causative mutation. We used InDel markers (Woods et al., 2014) to locate the mutation within a 2 Mb region on chromosome 2 (Figure 1C). Analysis of candidate genes in the Bd21-3 genome revealed that this genomic region contained a homolog of *EARLY FLOWERING 3* (Bradi2g14290). Because mutations in this gene were known to cause rapid flowering in other species (Hicks et al., 1996, 2001; Zagotta et al., 1996; Faure et al., 2012; Yang et al., 2013; Boden et al., 2014; Alvarez et al., 2016; Rubenach et al., 2017), we sequenced the coding region of *ELF3* and found a single base pair mutation in the fourth exon of the gene that resulted in a premature STOP codon (Figure 1D) and is predicted to result in a truncated protein that lacks the fourth conserved domain of ELF3 (Figure 1E). The genotyping of a segregating population originating from a cross of the *elf3* mutant using dCAPS primers showed that the segregation of the phenotype was 100% linked with the presence of the *elf3* mutation (Supplementary Figure 2). To further confirm that the *elf3* mutation is causative, we were able to rescue the rapid flowering mutant phenotype by overexpressing the *ELF3* cDNA using the maize ubiquitin promoter (*UBI:ELF3*) in the *elf3* mutant background (Supplementary Figure 3). The mutant was back-crossed twice with Bd21-3 before further characterization.

### The *elf3* Phenotypes Mimic Long-Day Grown Plants

**FIGURE 2:**
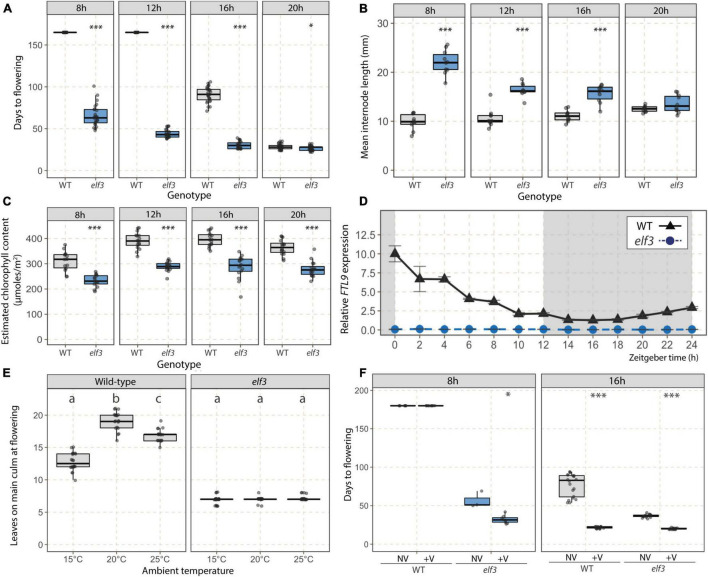
Phenotypic characterization of the *elf3* mutant. Flowering time (**A**; days to flowering), mean internode lengths **(B)**, and estimated chlorophyll contents (**C**; μmoles.m^–2^) for the *elf3* mutant wild-type (WT) plants under 8, 12, 16, and 20 h photoperiods (*n* = 10–25). For **(B,C)**, data were collected 1 week prior to estimated floral transition according to a preliminary experiment. **(D)** Relative *FTL9* expression kinetics in WT (black triangles) and *elf3* (blue circles) plants under a 12 h photoperiod. Data were normalized using the geometric mean of *ACT3* and *UBC18* reference genes. Error bars represent the standard error of the mean. Time is expressed as the number of hours following the start of the light period (Zeitgeber time, ZT). **(E)** Flowering time (number of leaves on the main culm at flowering) of *elf3* mutant and WT plants grown at distinct temperatures. Letters (a,b,c) indicate statistical differences (*p* < 0.05) according to a Tukey’s HSD test used to perform multiple comparisons. **(F)** Flowering time (days to flowering) of *elf3* mutant and WT plants exposed (V) or not (NV) to a 3-week vernalizing treatment and subsequently grown under 8 or 16 h photoperiods. Student *t*-tests were used for pairwise comparisons in **(A–C,F)** (^***^p < 0.001; *p < 0.05).

We further characterized the *elf3* mutant by growing it under 8, 12, 16, and 20 h photoperiods. The mutant flowered earlier than WT plants under all photoperiods except 20 h, under which both genotypes flowered very rapidly (Figure 2A). We also observed increased internode lengths (Figure 2B) and a lower estimated chlorophyll content in the *elf3* mutant (Figure 2C). The difference in mean internode lengths was dependent on the photoperiod as the length of internodes increased with longer photoperiods in WT plants and decreased in *elf3* plants, such that no difference was measured under 20 h photoperiod. The estimated chlorophyll content, on the contrary, was significatively lower in the mutant under all photoperiods, which is in accordance with the color of the leaves that were visibly paler in the mutant. Because increased RNA level of *FT-Like9* (*FTL9*) was shown to be a marker of SD in *B. distachyon* (Qin et al., 2019; Woods et al., 2019), we tested whether its expression was altered in the mutant under a 12 h photoperiod. We observed that *FTL9* transcripts were undetectable at all-time points in the *elf3* mutant under conditions in which the gene was highly expressed in Bd21-3 (Figure 2D).

We next tested the effect of temperature on the flowering time of the *elf3* mutant. Both higher (25°C) and lower (15°C) ambient temperatures accelerated flowering of WT plants grown under a 16 h photoperiod compared to the 20°C standard in terms of leaf number on the primary culm (Figure 2E). In contrast, the number of leaves at flowering was not altered by temperature changes in the *elf3* mutant (Figure 2E). We then tested the effect of vernalization on the *elf3* mutant by exposing seeds to 3 weeks of cold (4°C; a saturating vernalization treatment for Bd21-3, as shown in Ream et al., 2014) before growing them in either 8 or 16 h photoperiods (Figure 2F). The early flowering of *elf3* was further accelerated by vernalization under both photoperiods, suggesting that the mutation of *ELF3* does not affect the vernalization response. Because vernalization provides the competence to flower through the up-regulation of *VRN1* in *B. distachyon* (Ream et al., 2014), we tested whether elevated *VRN1* mRNA levels affected the flowering time of the *elf3* mutant without vernalization. We found that homozygous lines overexpressing *VRN1* (*UBI:VRN1* lines originally developed and characterized in Ream et al., 2014) in the *elf3* mutant background displayed an even more rapid flowering phenotype than the *elf3* mutant (Supplementary Figure 4).

### Role of EARLY FLOWERING 3 in Controlling Circadian Clock and Flowering Time Genes

**FIGURE 3:**
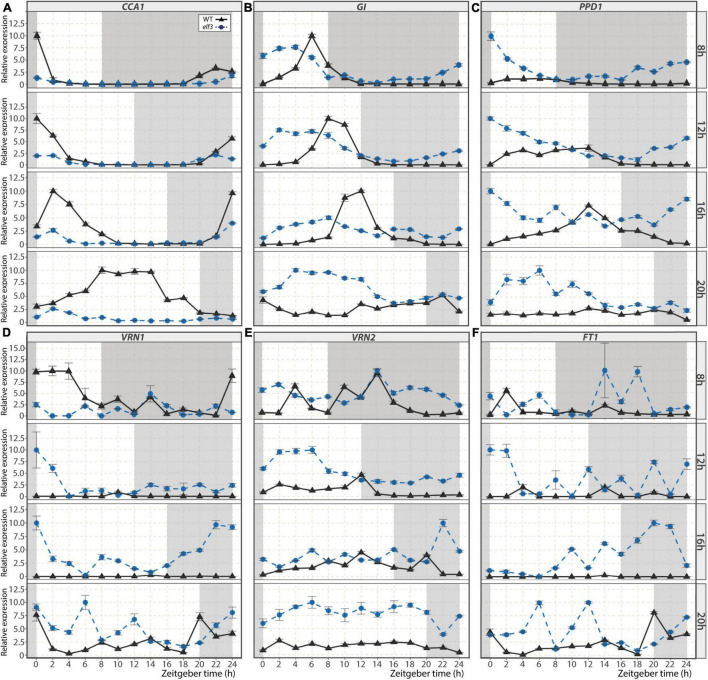
Expression kinetics of selected genes in the *elf3* mutant. Relative expression of circadian clock-regulated genes *CCA1* **(A)**, *GI* **(B)**, *PPD1* **(C)**, and the flowering genes *VRN1* **(D)**, *VRN2* **(E)**, and *FT1* **(F)** during a 24 h time course for WT plants (black triangles) and *elf3* mutants (blue circle) cultivated under 8, 12, 16, and 20 h photoperiods. Error bars represent the standard error of the mean. Data were normalized using the geometric mean of *ACT3* and *UBC18*. The white background indicates the light period, and the gray background shows the dark period. Time is expressed as the number of hours following the start of the light period (Zeitgeber time, ZT).

The ELF3 protein is a component of the EC of the circadian clock, which controls the expression of other clock genes, as well as flowering genes (Huang and Nusinow, 2016). We thus analyzed the expression patterns of a set of those targets in the leaves of the *elf3* mutant under different photoperiods (Figure 3). In WT plants, the peak of *CCA1* expression occurs in the morning in all photoperiods, except under 20 h LD, in which it occurs at midday (Figure 3A). In the *elf3* mutant, these peaks were strongly damped under all photoperiods, indicating that ELF3 is required for proper induction of *CCA1* expression. Alterations were also visible in the expression kinetics of *GI*, another clock gene that participates in Arabidopsis flowering induction (Figure 3B). The expression peak of *GI* was advanced by about 4 h in the *elf3* mutant under all photoperiods. Interestingly, whereas *GI* expression was undetectable during the nights under photoperiods shorter than 20 h in WT plants, it could be detected at most time points in the *elf3* mutant independently of the photoperiod. The expression of *PPD1*, a clock-regulated flowering time regulator in temperate grasses (Turner et al., 2005), was also altered: it was much stronger at all-time points in the *elf3* mutant except at the end of the day when the expression level was similar to that in WT (Figure 3C). On the contrary, the expression of *CO1*, another output of the circadian clock (Shaw et al., 2020), seemed to be downregulated at night in the mutant under photoperiods shorter than 20 h LD (Supplementary Figure 5). Overall, these results show the strong impact that mutation of *ELF3* has on circadian clock-regulated gene expression in *B. distachyon*.

We then analyzed the expression patterns of the flowering time genes *VRN1* and *VRN2*. Although the expression of the floral inducer *VRN1* seemed slightly down-regulated in the *elf3* mutant under 8 h SD during daytime, we observed an increase in its expression in all other photoperiods (Figure 3D). Interestingly, the expression of the floral repressor *VRN2* was also stimulated at most time points in the mutant (Figure 3E). Because *VRN1* and *VRN2* play antagonistic roles in controlling the expression of the florigen *FT1* (Woods et al., 2016) and were both up-regulated in *elf3*, we examined *FT1* expression. Consistent with the rapid flowering *elf3* phenotype, *FT1* expression was higher in the mutant than in WT plants (Figure 3F).

### Links Between EARLY FLOWERING 3, Photoreceptors, and Night Breaks

**FIGURE 4:**
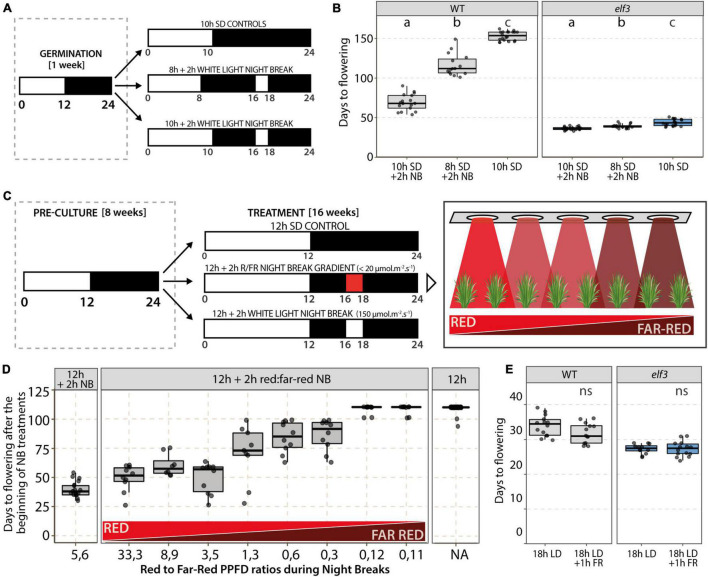
Effect of night breaks and light quality on flowering time. **(A)** Experimental design and **(B)** results of night break (NB) experiments performed by exposing wild-type (WT) and *elf3* plants growing in either 8 or 10 h photoperiods to a 2 h NB (NB; 150 μmol.m^–2^.s^–1^) from Zeitgeber time (ZT) 16 h to ZT18h. Controls were maintained in 10 h SD (*n* = 15–20). Note that most of the control plants had not flowered after 150 days in 10 h SD. Letters indicate statistical difference (*p* < 0.05) using Tukey’s HSD tests. **(C)** Experimental design used to test the effect of the red (R) to far-red (FR) ratio during NBs on flowering. **(D)** Flowering time of plants exposed to a NB under a red to far-red gradient. WT plants were grown in a 12 h photoperiod for 8 weeks before being transferred to a 12 h photoperiod supplemented with a 2 h NB (from ZT16h to ZT18h) under a red to far-red gradient (*n* = 8–15 per condition). Control plants were either maintained under a 12 h photoperiod (Right panel) or exposed to a 2 h white light NB (Left panel). **(E)** Effect of far-red (FR) light on flowering time. At the beginning of the night, WT and *elf3* plants grown under an 18 h photoperiod were exposed to 1 h of FR light (25 μmol.m^–2^.s^–1^). Control plants were exposed to an uninterrupted night (*n* = 15–20); *t*-test revealed no statistically significant differences (ns).

To test whether night breaks (NBs) could accelerate flowering in the *elf3* mutant as in WT plants, we exposed plants grown in 8 or 10 h SD to a 2 h NB from ZT16h to ZT18h (Figure 4A). While most of the control plants had not flowered after 150 days, the exposure to NBs accelerated flowering of WT plants both in 8 h SD, in which plants flowered 100–125 days after germination, and even more in 10 h SD, in which they flowered after around 70 days (Figure 4B). In the *elf3* mutant, we also observed a very slight acceleration of flowering upon NB exposure—about 4 days when NBs were provided in 8 h SD and 7 days under 10 h SD—indicating that the *elf3* mutation attenuates the flowering response to NB.

We then decided to test the effect of varying red to far-red ratios on the NB efficiency. Accordingly, wild-type plants grown under 12 h SD for 8 weeks were exposed daily to a 2 h NB from ZT16h to ZT18h, provided as a mixture of red and far-red light (Figure 4C). Different red:far-red ratios were provided using an LED light gradient during the NB, with low light intensities to limit photosynthetic effects. Control plants were either maintained under SD without NB or exposed to 2 h NBs of white light. We observed a strong correlation between flowering induction and higher red:far-red ratios. Indeed, red:far-red ratios over 3 led to the strongest acceleration of flowering, whereas ratios between 0.3 and 1.3 provided only a slight acceleration of flowering, and lower ratios did not induce flowering (Figure 4D). Finally, we performed end-of-day far-red treatments to see whether switching phytochromes to their inactive Pr form before night-time would affect flowering. We therefore provided a 1-h far-red treatment to plants grown under 18 h LD and observed that the far-red treatment at the end of the day did not affect flowering time (Figure 4E).

## Discussion and Perspectives

### Interplay Between EARLY FLOWERING 3, the Photoperiod, and the Circadian Clock

Although much is known about the many genes whose mutations affect flowering in Arabidopsis (Bouché et al., 2016), substantially fewer flowering control genes have been identified to date in temperate grasses, and many of these genes do not have homologs involved in flowering in Arabidopsis (Higgins et al., 2010; Ream et al., 2012). However, *ELF3* has been described as a key hub between photoperiodic signals and the circadian clock in both eudicots and monocots (Huang and Nusinow, 2016) and the results presented in this paper strongly support this interpretation and extend the characterization of the role of *ELF3* to *B. distachyon*. Indeed, a mutation in *ELF3* severely reduces the requirement for LD exposure to induce flowering in the Bd21-3 accession, similar to the phenotype described in a preprint from Gao et al. (2019).

That flowering in the *elf3* mutant occurs rapidly in all photoperiods including 8 h SD indicates that *elf3* mutants perceive all photoperiods as LD. In some plants, including *B. distachyon* and other temperate grasses, the exposure to the shorter photoperiods of winter can substitute for the exposure to cold temperature as a winter cue providing floral competence, a process known as SD vernalization (Purvis and Gregory, 1937). Recently, *FTL9* was shown to be key in establishing the SD-vernalization ability of *B. distachyon*, and *FTL9* transcript levels exhibit a diurnal oscillation with a high peak in SD but are always low in LD (Woods et al., 2019). We observed that the expression of *FTL9* was undetectable in the *elf3* mutant in SD throughout a diurnal cycle. Interestingly, this expression pattern is opposite to that observed in the late flowering *phyC* mutant, in which flowering is insensitive to LD (Woods et al., 2014, 2019). Therefore, the disruption of the EC complex caused by the absence of functional ELF3 mimics constitutive exposure to LD like the absence of phyC mimics constitutive exposure to SD. Furthermore, these mutants show opposite transcriptomic profiles for several gene clusters (Gao et al., 2019). The specific pathways through which ELF3 and phyC exert their opposite roles remain to be determined. ELF3 might also act through FT1-independent pathways; for example, in Arabidopsis, the *elf3*;*co* double mutant is early flowering but does not display any increase in *FT* expression (Kim et al., 2005; Song et al., 2018). In *B. distachyon*, it was shown that the *phyC* mutant does not display any difference in *ELF3* expression (Woods et al., 2014), but a large part of the regulation of ELF3 function occurs at the protein level (Huang and Nusinow, 2016). The physical interaction between ELF3 and PHYC, which has been reported (Gao et al., 2019), could thus be critical in the regulatory process.

### Interactions Between the *elf3* Mutation and Other Flowering Pathways

The *elf3* mutant was found to be insensitive to SD but still responded to vernalization by cold. Indeed, a 3-week exposure to 4°C further accelerated the flowering of the mutant in all tested photoperiods. At the molecular level, we found that the expression of the positive regulator of flowering *VRN1* was low under 8 h SD in the *elf3* mutant, which seems in contrast with its rapid flowering phenotype. However, the rapid flowering but low *VRN1* RNA levels could be caused by the increase in the expression of *PPD1* under SD that we observed in the mutant. Indeed, in wheat, the *ppd1* mutation causes increased *VRN1* mRNA levels specifically under short photoperiods, indicating that PPD1 is a negative regulator of *VRN1* under SD (Shaw et al., 2020); thus, the increased *PPD1* expression levels in the *elf3* mutant in SD could be responsible for the observed repression of *VRN1*. The *VRN1*-independent acceleration of flowering in *elf3* could also be due to the increase in *PPD1* which is itself a flowering promoter (Shaw et al., 2013, 2020). It is noteworthy that the *elf3* mutant is responsive to *VRN1* because the overexpression of *VRN1* in the *elf3* background results in a very rapid flowering under SD, highlighting the additive roles played by the vernalization and the photoperiodic pathways. In 16 h LD, we found higher *VRN1* expression levels in the *elf3* background in the absence of cold. However, the vernalization treatment also accelerated flowering in the *elf3* mutant under 16 h LD, suggesting that cold exposure accelerates flowering either by further activation of *VRN1* or by the regulation of other targets. Further experiments are required to test these possibilities.

Ambient temperature also plays a role in flowering time control in many species (Capovilla et al., 2015). In *B. distachyon*, earlier reports showed that increasing the ambient temperature cannot substitute for LD to induce flowering (Boden et al., 2013), and that different accessions have distinct optimal temperatures for floral induction (Li et al., 2019). In our conditions, Bd21-3 flowered much more rapidly at 15°C than at 20 or 25°C. A similar observation was made in winter wheat cultivars, in which bolting occurred earlier at 11°C than at 25°C (Dixon et al., 2019). However, we did not see any effect of ambient temperature on the flowering time of the *elf3* mutant. This lack of temperature-response might either be due to the rapid flowering phenotype of the mutant, which would mask the temperature effect, or could indicate that *ELF3* plays a role in the temperature-dependent flowering response, as suggested earlier in barley (Ford et al., 2016). In Arabidopsis, the ELF3 protein acts as a thermosensor: at high temperature it is sequestered in liquid droplets and is prevented from exerting its transcriptional repressor role, resulting in accelerated flowering (Jung et al., 2020). However, the prion-like domain required for this behavior is absent in the *B. distachyon* ELF3 protein (Jung et al., 2020); moreover, the acceleration of flowering in the Bd21-3 accession is observed at lower rather than higher temperatures as in Arabidopsis. Because phyC and ELF3 proteins were shown to interact physically (Gao et al., 2019), one hypothesis would be that changes in ambient temperatures affect their interaction to modulate the repressing effect of ELF3 on the phyC-mediated induction of flowering. It would be interesting to test whether natural variation in *ELF3*, *phyC*, and *PIF*s among *B. distachyon* accessions affects temperature sensitivity for flowering induction.

### Perception of the Photoperiodic Pathway

The pathways through which photoperiodic signals are perceived and implemented into developmental responses in temperate grasses are not fully understood, and NB experiments can shed light on underlying mechanisms. Consistent with a previous study (Gao et al., 2019), we observed that the exposure of Bd21-3 plants to NBs was sufficient to accelerate flowering in SD. In wheat, the induction of flowering can also be triggered by NBs provided at different time points to plants grown in SD, and this acceleration of flowering was shown to require a functional *PPD1* allele (Pearce et al., 2017). When applying daily NBs to the *elf3* mutant, we observed only a very slight acceleration of flowering, suggesting that NBs act mainly through ELF3-mediated processes, although parallel pathways might also play a minor role, possibly through *GI*, *CO*, or yet unknown pathways. Further molecular work is required to establish the pathway that is triggered under such conditions.

Phytochromes can switch between the inactive Pr form, which accumulates under darkness or far-red light, and the active Pfr form, which is stimulated by red light (Quail, 2002). In Arabidopsis, lower red:far-red ratios, which indicate the presence of proximate plants that compete for light exposure, results in the acceleration of flowering (Casal, 2013). On the contrary, in wheat, lower red:far-red ratios were shown to reduce yields through delayed spike development and reduced floret numbers (Ugarte et al., 2010). Here we provided NBs using a varying mixture of low intensity red and far-red lights to *B. distachyon* plants grown under 12 h non-inductive conditions, and we observed a positive correlation between the induction of flowering and higher red:far-red ratios. These results suggest that phytochromes in their Pfr form stimulate floral induction although they do not preclude the participation of other molecular pathways in the induction of flowering.

The promotion of flowering by NBs supports the external coincidence model of the photoperiodic control of flowering in which the inductive pathways are activated when light is perceived at the appropriate circadian time. Flowering of LD plants can indeed be induced without increasing the length of the photoperiod but by displacing SD at the appropriate circadian time. These “displaced SD” can be reduced in length and still induce flowering, as shown for example in *Lolium temulentum* (Périlleux et al., 1994). An alternative explanation was proposed in which the photoperiod-mediated induction of flowering in temperate grasses relies on the hourglass model (Borthwick and Hendricks, 1960). In this model, the effect of LD is due to the shorter nights that do not allow a full reversion of the pool of active Pfr into the inactive Pr form, so that flowering is eventually triggered by the accumulation of the active Pfr form. However, a previous report in wheat indicated that far-red light, which induces full reversion of Pfr into Pr, diminishes the effect of 1 h NBs when given during the NB but not after, indicating that 1 h NBs are sufficient to irreversibly activate flowering (Pearce et al., 2017). Here, in *B. distachyon*, we showed that when exposing the WT and the *elf3* mutant to a 1 h far-red treatment at the end of each 18 h photoperiod, flowering was not delayed. Collectively, these results suggest that neither the day-to-day accumulation of active Pfr nor its role during night-time are key to floral induction, and rather indicate that Pfr plays its inductive role before the end of the light period in LD. Complementary experiments using transgenics constitutively expressing active phytochromes or experimental designs in which far-red light is provided during the daytime to reduce the accumulation of the active Pfr would help to further elucidate the underlying mechanisms. Recently, the introduction of a constitutively active, light-insensitive, allele of the rice phyB into *B. distachyon* led to a mild acceleration (4 days) of flowering in 16 h LD grown plants (Hu et al., 2020). The lack of a strong rapid flowering phenotype in these transgenics might be the indirect consequence of phyB mode of action, which could require the formation of an heterodimer with limiting levels of active phyC, or due to the heterologous rice phytochrome being used instead of the *B. distachyon* phytochrome. However, testing whether flowering is also accelerated under non-inductive photoperiods and whether these transgenics remain sensitive to NB would provide valuable insights. In any case, the current knowledge acquired through both physiological and transgenic experiments indicates that the external coincidence model does play a role in the photoperiodic induction of flowering in temperate grasses.

### Perspectives on the Photoperiodic Research in *Brachypodium distachyon*

**FIGURE 5:**
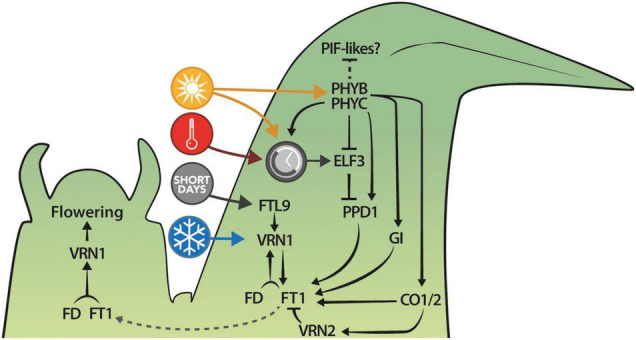
Summary of the current understanding of the photoperiodic regulation of flowering in *B. distachyon*. Colored circles represent the ambient temperature (red), the photoperiod (yellow), the circadian clock (gray), and the vernalization (blue) pathways.

The current model of the photoperiodic induction of flowering in *B. distachyon* involves the phyC-mediated activation of *PPD1* expression, possibly in part through *ELF3* (Figure 5). In turn, PPD1 induces the expression of *FT1* in leaves, which forms a positive regulatory loop with *VRN1* before FT1 protein moves toward the shoot apical meristem to induce flowering (Woods and Amasino, 2015). The elucidation of the exact pathway—or pathways—triggering flowering, however, will require more genetic work, including the creation of multiple mutants and transgenic lines, and the new mutant allele of *ELF3* described here provides an additional tool toward this goal. For example, *phyC;elf3* or *phyB;elf3* double mutants would be informative to evaluate if indeed most of the light signal from the phytochromes is mediated through ELF3. Furthermore, ELF3 plays a repressive role on *PPD1* but is *PPD1* the main target impacting photoperiodic flowering or is ELF3 involved in repressing other important components of the photoperiod pathway? The *elf3;ppd1* double mutants would be well suited to address this important question. Also, coupling these lines with mutants and overexpressors for *GI, CO1*, and *CO2* will help to test the epistatic interactions between these genes as well as their involvement in the photoperiodic pathway of floral induction. Indeed, exploring the impact these genes have on flowering has already led to some insights. For example, knock-down of *CO1* in *B. distachyon via* RNAi results in lower *VRN2* mRNA levels yet plants are delayed in flowering, whereas overexpression of *CO1* results in higher *VRN2* mRNA levels but interestingly more rapid flowering (Qin et al., 2019). This is consistent with studies from barley where the overexpression of *HvCO1/CO2* results in more rapid flowering even though *HvVRN2* is elevated (Mulki and von Korff, 2015). However, in barley, when comparing *UBI:HvCO2* lines in a segregating population with and without *HvVRN2*, plants with a functional *HvVRN2* allele are more delayed in flowering than those where *HvVRN2* is deleted, despite the presence of *UBI:HvCO2* in both segregating plants (Mulki and von Korff, 2015). The studies above highlight the importance of comparative genetic studies which, together with the development of new tools for *B. distachyon* (e.g., tissue-specific promoters, interactome maps, etc.), will help us to decipher the spatio-temporal regulation of flowering time in temperate grasses.

Finally, the recent improvements in LED technology will help to better understand the genetic regulations occurring in natural environments. Often, the lighting and temperature conditions used to grow plants in culture chambers do not reflect actual outdoor conditions. In *A. thaliana*, the expression of florigen/*FT* shows a different pattern in the field— with a peak in the morning—than previously described in the literature (Song et al., 2018). Exposing plants to daily temperature rhythms as well as changing red:far-red ratios in growth chambers was sufficient to mimic its natural expression pattern (Song et al., 2018). In *B. distachyon*, most of the diurnal gene regulation is caused by changes in the ambient temperature rather than light (Matos et al., 2014; MacKinnon et al., 2020), and phytochromes are known to act as thermosensors as well as photoreceptors (Franklin et al., 2014). Custom LED lighting systems associated with phytotronic cabinets now provide the possibility to better reproduce daily and seasonal cycles of temperature and daylight spectrum in any region of the planet (Wilson et al., 2015), thus opening new areas of exploration regarding the genetic mechanisms governing the adaptation to local environments, an evolutionary process to which *ELF3* could be key.

## Data Availability Statement

The raw data supporting the conclusions of this article will be made available by the authors, without undue reservation.

## Author Contributions

FB, DW, RA, and CP contributed to conception and design of the study. DW, FB, and RA performed the mutagenesis experiment, the screening, and the mapping of *elf3* mutant. FB and JL performed the expression analyses and the night break experiments. WL and KM produced plant material for genetic analyses. FB wrote the first draft of the manuscript. DW, WL, RA, and CP contributed to the submitted version of the manuscript. All authors read and approved the submitted version.

## Conflict of Interest

The authors declare that the research was conducted in the absence of any commercial or financial relationships that could be construed as a potential conflict of interest.

## Publisher’s Note

All claims expressed in this article are solely those of the authors and do not necessarily represent those of their affiliated organizations, or those of the publisher, the editors and the reviewers. Any product that may be evaluated in this article, or claim that may be made by its manufacturer, is not guaranteed or endorsed by the publisher.

## Supplementary Material

Supplementary Figure 1 Spectra and light conditions records. **(A)** Spectral distribution of white, red, and far-red lights used during night breaks and end-of-day far-red treatments. **(B)** Diurnal monitoring of far-red, total light, and photosynthetically active radiations (PAR) intensities for plants exposed to 18 h long day (LD) followed by 1 h end-of-day far-red (FR) treatment (left) or not (right).

Supplementary Figure 2 Example of a Derived Cleaved Amplified Polymorphic Sequence for *elf3* genotyping. The PCR was performed using the dCAPS primer pair shown in Supplementary Table 1. The PCR product was digested using the hpy166ii restriction enzyme (NEB, United States), which cuts only into the WT (+) sequences, creating shorter fragments than in mutated *ELF3* sequences (–). The *elf3* mutant phenotypes segregated perfectly with the homozygous *elf3* mutation.

Supplementary Figure 3 Complementation of *elf3* mutant with the *UBI:ELF3* construct. Six independent transgenic lines exhibited rescue of the rapid flowering phenotype in the T0 generation under 16-h LD. Controls were sown 3 weeks before transplantation of T0 lines to soil. The picture was taken 45 days after transplantation to soil.

Supplementary Figure 4 Flowering phenotype of *elf3* mutants overexpressing *VRN1*. Individuals were segregated from the *elf3*;*UBI*:*VRN1* F2 population. The picture was taken 110 days after germination under 8 h SD. *elf3* mutant flowered after 86.5 ± 13.7 days (*n* = 15), *elf3*;*UBI*:*VRN1* flowered after 36.3 ± 2.9 days (*n* = 27). WT and *UBI:VRN1* plants had not flowered after 180 days.

Supplementary Figure 5 Expression kinetics of *CO1* in the *elf3* mutant. Relative expression of *CO1* during a 24 h time course for WT plants (black triangles) and *elf3* mutants (blue circle) cultivated under 8, 12, 16, and 20 h photoperiods. Error bars represent the standard error of the mean. Data were normalized using the geometric mean of *ACT3* and *UBC18*. The white background indicates the light period, and the gray background shows the dark period. Time is expressed as the number of hours following the start of the light period (Zeitgeber time, ZT).

## References

[B1] Alvarez M. A. Tranquilli G. Lewis S. Kippes N. Dubcovsky J. (2016). Genetic and physical mapping of the earliness per se locus Eps-Am1 in *Triticum monococcum* identifies *EARLY FLOWERING 3* (*ELF3*) as a candidate gene. *Funct. Integr. Genomics* 16 365–382. 10.1007/s10142-016-0490-3 27085709 PMC4947483

[B2] An H. Roussot C. Suárez-López P. Corbesier L. Vincent C. Piñeiro M. (2004). CONSTANS acts in the phloem to regulate a systemic signal that induces photoperiodic flowering of *Arabidopsis*. *Development* 131 3615–3626. 10.1242/dev.01231 15229176

[B3] Bendix C. Marshall C. M. Harmon F. G. (2015). Circadian clock genes universally control key agricultural traits. *Mol. Plant* 8 1135–1152. 10.1016/j.molp.2015.03.003 25772379

[B4] Boden S. A. Kavanová M. Finnegan E. J. Wigge P. A. (2013). Thermal stress effects on grain yield in *Brachypodium distachyon* occur *via* H2A.Z-nucleosomes. *Genome Biol.* 14:R65. 10.1186/gb-2013-14-6-r65 23800039 PMC4062847

[B5] Boden S. A. Weiss D. Ross J. J. Davies N. W. Trevaskis B. Chandler P. M. (2014). *EARLY FLOWERING 3* regulates flowering in spring barley by mediating gibberellin production and flowering locus T expression. *Plant Cell* 26 1557–1569. 10.1105/tpc.114.123794 24781117 PMC4036571

[B6] Borthwick H. A. Hendricks S. B. (1960). Photoperiodism in plants. *Science* 132 1223–1228. 10.1126/science.132.3435.1223 17801667

[B7] Bouché F. Lobet G. Tocquin P. Périlleux C. (2016). FLOR-ID: an interactive database of flowering-time gene networks in Arabidopsis thaliana. *Nucleic Acids Res.* 44 D1167–D1171. 10.1093/nar/gkv1054 26476447 PMC4702789

[B8] Bu T. Lu S. Wang K. Dong L. Li S. Xie Q. (2021). A critical role of the soybean evening complex in the control of photoperiod sensitivity and adaptation. *Proc. Natl. Acad. Sci. U.S.A.* 118:e2010241118. 10.1073/pnas.2010241118 33558416 PMC7923351

[B9] Bullrich L. Appendino M. Tranquilli G. Lewis S. Dubcovsky J. (2002). Mapping of a thermo-sensitive earliness per se gene on *Triticum monococcum* chromosome 1Am. *Theor. Appl. Genet.* 105 585–593. 10.1007/s00122-002-0982-5 12582508

[B10] Bunning E. (1937). Die endonome tagesrhythmik als grundlage der photoperiodischen reaktion. *Ber. Deut. Bot. Ges.* 54 590–607.

[B11] Capovilla G. Schmid M. Posé D. (2015). Control of flowering by ambient temperature. *J. Exp. Bot.* 66 59–69. 10.1093/jxb/eru416 25326628

[B12] Casal J. J. (2013). Photoreceptor signaling networks in plant responses to shade. *Annu. Rev. Plant Biol.* 64 403–427. 10.1146/annurev-arplant-050312-120221 23373700

[B13] Casao M. C. Karsai I. Igartua E. Gracia M. P. Veisz O. Casas A. M. (2011). Adaptation of barley to mild winters: a role for PPDH2. *BMC Plant Biol.* 11:164. 10.1186/1471-2229-11-164 22098798 PMC3226555

[B14] Chen A. Li C. Hu W. Lau M. Y. Lin H. Rockwell N. C. (2014). Phytochrome C plays a major role in the acceleration of wheat flowering under long-day photoperiod. *Proc. Natl. Acad. Sci. U.S.A.* 111 10037–10044. 10.1073/pnas.1409795111 24961368 PMC4104863

[B15] Cheng M.-C. Kathare P. K. Paik I. Huq E. (2021). Phytochrome signaling networks. *Annu. Rev. Plant Biol.* 72 217–244. 10.1146/annurev-arplant-080620-024221 33756095 PMC10988782

[B16] Corbesier L. Vincent C. Jang S. Fornara F. Fan Q. Searle I. (2007). FT protein movement contributes to long-distance signaling in floral induction of *Arabidopsis*. *Science* 316 1030–1033. 10.1126/science.1141752 17446353

[B17] Covington M. F. (2001). ELF3 modulates resetting of the circadian clock in *Arabidopsis*. *Plant Cell Online* 13 1305–1316. 10.1105/tpc.13.6.1305 11402162 PMC135573

[B18] Covington M. F. Maloof J. N. Straume M. Kay S. A. Harmer S. L. (2008). Global transcriptome analysis reveals circadian regulation of key pathways in plant growth and development. *Genome Biol.* 9:R130. 10.1186/gb-2008-9-8-r130 18710561 PMC2575520

[B19] Creux N. Harmer S. (2019). Circadian rhythms in plants. *Cold Spring Harb. Perspect. Biol.* 11:a034611. 10.1101/cshperspect.a034611 31138544 PMC6719598

[B20] Distelfeld A. Dubcovsky J. (2010). Characterization of the maintained vegetative phase deletions from diploid wheat and their effect on VRN2 and FT transcript levels. *Mol. Genet. Genom.* 283 223–232. 10.1007/s00438-009-0510-2 20063107 PMC2820692

[B21] Dixon L. E. Karsai I. Kiss T. Adamski N. M. Liu Z. Ding Y. (2019). Vernalization1 controls developmental responses of winter wheat under high ambient temperatures. *Development* 146:dev172684. 10.1242/dev.172684 30770359 PMC6382010

[B22] Doyle M. R. Davis S. J. Bastow R. M. McWatters H. G. Kozma-Bognár L. Nagy F. (2002). The ELF4 gene controls circadian rhythms and flowering time in *Arabidopsis thaliana*. *Nature* 419 74–77. 10.1038/nature00954 12214234

[B23] Faure S. Turner A. S. Gruszka D. Christodoulou V. Davis S. J. von Korff M. (2012). Mutation at the circadian clock gene early maturity 8 adapts domesticated barley (Hordeum vulgare) to short growing seasons. *Proc. Natl. Acad. Sci. U.S.A.* 109 8328–8333. 10.1073/pnas.1120496109 22566625 PMC3361427

[B24] Ford B. Deng W. Clausen J. Oliver S. Boden S. Hemming M. (2016). Barley (*Hordeum vulgare*) circadian clock genes can respond rapidly to temperature in an *EARLY FLOWERING 3* -dependent manner. *J. Exp. Bot.* 67 5517–5528. 10.1093/jxb/erw317 27580625 PMC5049398

[B25] Franklin K. A. Toledo-Ortiz G. Pyott D. E. Halliday K. J. (2014). Interaction of light and temperature signalling. *J. Exp. Bot.* 65 2859–2871. 10.1093/jxb/eru059 24569036

[B26] Gao M. Geng F. Klose C. Staudt A.-M. Huang H. Nguyen D. (2019). Phytochromes measure photoperiod in *Brachypodium*. *bioRxiv* [Preprint]. 10.1101/697169

[B27] Goodstein D. M. Shu S. Howson R. Neupane R. Hayes R. D. Fazo J. (2012). Phytozome: a comparative platform for green plant genomics. *Nucleic Acids Res.* 40 D1178–D1186. 10.1093/nar/gkr944 22110026 PMC3245001

[B28] Goto N. Kumagai T. Koornneef M. (1991). Flowering responses to light-breaks in photomorphogenic mutants of *Arabidopsis thaliana*, a long-day plant. *Physiol. Plant.* 83 209–215. 10.1111/j.1399-3054.1991.tb02144.x

[B29] Gustafsson Å Hagberg A. Lundqvist U. (1960). The induction of early mutants in bonus barley. *Hereditas* 46 675–699. 10.1111/j.1601-5223.1960.tb03109.x

[B30] Hazen S. P. Schultz T. F. Pruneda-Paz J. L. Borevitz J. O. Ecker J. R. Kay S. A. (2005). *LUX ARRYTHMO* encodes a MYB domain protein essential for circadian rhythms. *Proc. Natl. Acad. Sci. U.S.A.* 102 10387–10392. 10.1073/pnas.0503029102 16006522 PMC1177380

[B31] Hicks K. A. Albertson T. M. Wagner D. R. (2001). *EARLY FLOWERING 3* encodes a novel protein that regulates circadian clock function and flowering in *Arabidopsis*. *Plant Cell* 13 1281–1292. 10.1105/tpc.010070 11402160 PMC135582

[B32] Hicks K. A. Millar A. J. Carré I. A. Somers D. E. Straume M. Meeks-Wagner D. R. (1996). Conditional circadian dysfunction of the arabidopsis early-flowering 3 mutant. *Science* 274 790–792. 10.1126/science.274.5288.790 8864121

[B33] Higgins J. A. Bailey P. C. Laurie D. A. (2010). Comparative genomics of flowering time pathways using *Brachypodium distachyon* as a model for the temperate grasses. *PLoS One* 5:e10065. 10.1371/journal.pone.0010065 20419097 PMC2856676

[B34] Hu W. Figueroa-Balderas R. Chi-Ham C. Lagarias J. C. (2020). Regulation of monocot and dicot plant development with constitutively active alleles of phytochrome B. *Plant Direct* 4:e00210. 10.1002/pld3.210 32346668 PMC7184922

[B35] Huang H. Gehan M. A. Huss S. E. Alvarez S. Lizarraga C. Gruebbling E. L. (2017). Cross-species complementation reveals conserved functions for *EARLY FLOWERING 3* between monocots and dicots. *Plant Direct* 1: e00018. 10.1002/pld3.18 31245666 PMC6508535

[B36] Huang H. Nusinow D. A. (2016). Into the evening: complex interactions in the *Arabidopsis* circadian clock. *Trends Genet.* 32 674–686. 10.1016/j.tig.2016.08.002 27594171

[B37] Itoh H. Tanaka Y. Izawa T. (2018). Genetic relationship between phytochromes and OsELF3–1 reveals the mode of regulation for the suppression of phytochrome signaling in rice. *Plant Cell Physiol.* 60 549–561. 10.1093/pcp/pcy225 30476313

[B38] Johansson M. Staiger D. (2014). Time to flower: interplay between photoperiod and the circadian clock. *J. Exp. Bot.* 66 719–730. 10.1093/jxb/eru441 25371508

[B39] Jung J.-H. Barbosa A. D. Hutin S. Kumita J. R. Gao M. Derwort D. (2020). A prion-like domain in ELF3 functions as a thermosensor in *Arabidopsis*. *Nature* 585 256–260. 10.1038/s41586-020-2644-7 32848244

[B40] Kim W.-Y. Hicks K. A. Somers D. E. (2005). Independent roles for *EARLY FLOWERING 3* and *ZEITLUPE* in the control of circadian timing, hypocotyl length, and flowering time. *Plant Physiol.* 139 1557–1569. 10.1104/pp.105.067173 16258016 PMC1283789

[B41] Kippes N. VanGessel C. Hamilton J. Akpinar A. Budak H. Dubcovsky J. (2020). Effect of phyB and phyC loss-of-function mutations on the wheat transcriptome under short and long day photoperiods. *BMC Plant Biol.* 20:297. 10.1186/s12870-020-02506-0 32600268 PMC7325275

[B42] Legris M. Ince Y. Ç Fankhauser C. (2019). Molecular mechanisms underlying phytochrome-controlled morphogenesis in plants. *Nat. Commun.* 10:5219. 10.1038/s41467-019-13045-0 31745087 PMC6864062

[B43] Leivar P. Quail P. H. (2011). PIFs: pivotal components in a cellular signaling hub. *Trends Plant Sci.* 16 19–28. 10.1016/j.tplants.2010.08.003 20833098 PMC3019249

[B44] Li C. Dubcovsky J. (2008). Wheat FT protein regulates VRN1 transcription through interactions with FDL2. *Plant J.* 55 543–554. 10.1111/j.1365-313x.2008.03526.x 18433437 PMC4739743

[B45] Li M. Kennedy A. Huybrechts M. Dochy N. Geuten K. (2019). The effect of ambient temperature on *Brachypodium distachyon* development. *Front. Plant Sci.* 10:1011. 10.3389/fpls.2019.01011 31497030 PMC6712961

[B46] Lu S. Zhao X. Hu Y. Liu S. Nan H. Li X. (2017). Natural variation at the soybean J locus improves adaptation to the tropics and enhances yield. *Nat. Genet.* 49 773–779. 10.1038/ng.3819 28319089

[B47] Lucas M. Prat S. (2014). PIFs get BRright: phytochrome interacting factors as integrators of light and hormonal signals. *New Phytol.* 202 1126–1141. 10.1111/nph.12725 24571056

[B48] Lundqvist U. (2009). *Eighty Years of Scandinavian Barley Mutation Genetics and Breeding.* Rome: Food and Agriculture Organization of the United Nations.

[B49] MacKinnon K. J. Cole B. J. Yu C. Coomey J. H. Hartwick N. T. Remigereau M. (2020). Changes in ambient temperature are the prevailing cue in determining *Brachypodium distachyon* diurnal gene regulation. *New Phytol.* 227 1709–1724. 10.1111/nph.16507 32112414

[B50] Mathews S. (2010). Evolutionary studies illuminate the structural-functional model of plant phytochromes. *Plant Cell* 22 4–16. 10.1105/tpc.109.072280 20118225 PMC2828699

[B51] Matos D. A. Cole B. J. Whitney I. P. MacKinnon K. J.-M. Kay S. A. Hazen S. P. (2014). Daily changes in temperature, not the circadian clock, regulate growth rate in *Brachypodium distachyon*. *PLoS One* 9:e100072. 10.1371/journal.pone.0100072 24927130 PMC4057399

[B52] Matsubara K. Ogiso-Tanaka E. Hori K. Ebana K. Ando T. Yano M. (2012). Natural variation in Hd17, a homolog of *Arabidopsis* ELF3 that is involved in rice photoperiodic flowering. *Plant Cell Physiol.* 53 709–716. 10.1093/pcp/pcs028 22399582

[B53] Möglich A. Yang X. Ayers R. A. Moffat K. (2010). Structure and function of plant photoreceptors. *Annu. Rev. Plant Biol.* 61 21–47. 10.1146/annurev-arplant-042809-112259 20192744

[B54] Mulki M. A. von Korff M. (2015). Constans controls floral repression by up-regulating vernalization2 (VRN-H2) in Barley. *Plant Physiol.* 170 325–337. 10.1104/pp.15.01350 26556793 PMC4704585

[B55] Nieto C. López-Salmerón V. Davière J.-M. Prat S. (2015). ELF3-PIF4 interaction regulates plant growth independently of the evening complex. *Curr. Biol.* 25 187–193. 10.1016/j.cub.2014.10.070 25557667

[B56] Nishida H. Ishihara D. Ishii M. Kaneko T. Kawahigashi H. Akashi Y. (2013). Phytochrome C is a key factor controlling long-day flowering in Barley. *Plant Physiol.* 163 804–814. 10.1104/pp.113.222570 24014575 PMC3793059

[B57] Oakenfull R. J. Davis S. J. (2017). Shining a light on the *Arabidopsis* circadian clock. *Plant Cell Environ.* 40 2571–2585. 10.1111/pce.13033 28732105

[B58] Paik I. Huq E. (2019). Plant photoreceptors: multi-functional sensory proteins and their signaling networks. *Semin. Cell Dev. Biol.* 92 114–121. 10.1016/j.semcdb.2019.03.007 30946988 PMC8630751

[B59] Pearce S. Kippes N. Chen A. Debernardi J. M. Dubcovsky J. (2016). RNA-seq studies using wheat PHYTOCHROME B and PHYTOCHROME C mutants reveal shared and specific functions in the regulation of flowering and shade-avoidance pathways. *BMC Plant Biol.* 16:141. 10.1186/s12870-016-0831-3 27329140 PMC4915087

[B60] Pearce S. Shaw L. M. Lin H. Cotter J. D. Li C. Dubcovsky J. (2017). Night-break experiments shed light on the photoperiod1-mediated flowering. *Plant Physiol.* 174 1139–1150. 10.1104/pp.17.00361 28408541 PMC5462047

[B61] Périlleux C. Bernier G. Kinet J.-M. (1994). Circadian rhythms and the induction of flowering in the long-day grass *Lolium temulentum L*. *Plant Cell Environ.* 17 755–761. 10.1111/j.1365-3040.1994.tb00168.x

[B62] Purvis O. N. Gregory F. G. (1937). Studies in vernalisation of cereals: i. a comparative study of vernalisation of winter rye by low temperature and by short days. *Ann. Bot* 1 569–591. 10.2307/42906574

[B63] Qin Z. Bai Y. Muhammad S. Wu X. Deng P. Wu J. (2019). Divergent roles of FT-like 9 in flowering transition under different day lengths in *Brachypodium distachyon*. *Nat. Commun.* 10:812. 10.1038/s41467-019-08785-y 30778068 PMC6379408

[B64] Quail P. H. (2002). Phytochrome photosensory signalling networks. *Nat. Rev. Mol. Cell Bio.* 3 85–93. 10.1038/nrm728 11836510

[B65] Ream T. S. Woods D. P. Amasino R. M. (2012). The molecular basis of vernalization in different plant groups. *Cold Spring Harb. Symp. Quant Biol.* 77 105–115. 10.1101/sqb.2013.77.014449 23619014

[B66] Ream T. S. Woods D. P. Schwartz C. J. Sanabria C. P. Mahoy J. A. Walters E. M. (2014). Interaction of photoperiod and vernalization determines flowering time of *Brachypodium distachyon*. *Plant Physiol.* 164 694–709. 10.1104/pp.113.232678 24357601 PMC3912099

[B67] Rubenach A. J. S. Hecht V. Schoor J. K. V. Liew L. C. Aubert G. Burstin J. (2017). *EARLY FLOWERING 3* redundancy fine-tunes photoperiod sensitivity. *Plant Physiol.* 173 2253–2264. 10.1104/pp.16.01738 28202598 PMC5373058

[B68] Saito H. Ogiso-Tanaka E. Okumoto Y. Yoshitake Y. Izumi H. Yokoo T. (2012). Ef7 encodes an ELF3-like protein and promotes rice flowering by negatively regulating the floral repressor gene GHD7 under both short- and long-day conditions. *Plant Cell Physiol.* 53 717–728. 10.1093/pcp/pcs029 22422935

[B69] Seaton D. D. Toledo-Ortiz G. Ganpudi A. Kubota A. Imaizumi T. Halliday K. J. (2018). Dawn and photoperiod sensing by phytochrome A. *Proc. Natl. Acad. Sci. U.S.A.* 115:201803398. 10.1073/pnas.1803398115 30254157 PMC6187151

[B70] Shaw L. M. Li C. Woods D. P. Alvarez M. A. Lin H. Lau M. Y. (2020). Epistatic interactions between PHOTOPERIOD1, CONSTANS1 and CONSTANS2 modulate the photoperiodic response in wheat. *PLoS Genet.* 16:e1008812. 10.1371/journal.pgen.1008812 32658893 PMC7394450

[B71] Shaw L. M. Turner A. S. Herry L. Griffiths S. Laurie D. A. (2013). Mutant Alleles of Photoperiod-1 in Wheat (*Triticum aestivum L*.) that confer a late flowering phenotype in long days. *PLoS One* 8:e79459. 10.1371/journal.pone.0079459 24244507 PMC3828349

[B72] Song Y. H. Kubota A. Kwon M. S. Covington M. F. Lee N. Taagen E. R. (2018). Molecular basis of flowering under natural long-day conditions in *Arabidopsis*. *Nat. Plants* 4 824–835. 10.1038/s41477-018-0253-3 30250277 PMC6195122

[B73] Song Y. H. Shim J. S. Kinmonth-Schultz H. A. Imaizumi T. (2015). Photoperiodic flowering: time measurement mechanisms in leaves. *Annu. Rev. Plant Biol.* 66 441–464. 10.1146/annurev-arplant-043014-115555 25534513 PMC4414745

[B74] Strasser B. Alvarez M. J. Califano A. Cerdán P. D. (2009). A complementary role for ELF3 and TFL1 in the regulation of flowering time by ambient temperature. *Plant J.* 58 629–640. 10.1111/j.1365-313x.2009.03811.x 19187043

[B75] Suárez-López P. Wheatley K. Robson F. Onouchi H. Valverde F. Coupland G. (2001). CONSTANS mediates between the circadian clock and the control of flowering in *Arabidopsis*. *Nature* 410 1116–1120. 10.1038/35074138 11323677

[B76] Tamaki S. Matsuo S. Wong H. L. Yokoi S. Shimamoto K. (2007). Hd3a protein is a mobile flowering signal in rice. *Science* 316 1033–1036. 10.1126/science.1141753 17446351

[B77] Thines B. Harmon F. G. (2010). Ambient temperature response establishes ELF3 as a required component of the core *Arabidopsis* circadian clock. *Proc. Natl. Acad. Sci. U.S.A.* 107 3257–3262. 10.1073/pnas.0911006107 20133619 PMC2840299

[B78] Turner A. Beales J. Faure S. Dunford R. P. Laurie D. A. (2005). The pseudo-response regulator Ppd-H1 provides adaptation to photoperiod in Barley. *Science* 310 1031–1034. 10.1126/science.1117619 16284181

[B79] Ugarte C. C. Trupkin S. A. Ghiglione H. Slafer G. Casal J. J. (2010). Low red/far-red ratios delay spike and stem growth in wheat. *J. Exp. Bot.* 61 3151–3162. 10.1093/jxb/erq140 20497971 PMC2892155

[B80] Valverde F. Mouradov A. Soppe W. Ravenscroft D. Samach A. Coupland G. (2004). Photoreceptor regulation of constans protein in photoperiodic flowering. *Science* 303 1003–1006. 10.1126/science.1091761 14963328

[B81] Vandesompele J. Preter K. D. Pattyn F. Poppe B. Roy N. V. Paepe A. D. (2002). Accurate normalization of real-time quantitative RT-PCR data by geometric averaging of multiple internal control genes. *Genome Biol.* 3:research0034. 10.1186/gb-2002-3-7-research0034 12184808 PMC126239

[B82] Vogel J. Hill T. (2008). High-efficiency agrobacterium-mediated transformation of *Brachypodium distachyon* inbred line Bd21-3. *Plant Cell Rep.* 27 471–478. 10.1007/s00299-007-0472-y 17999063

[B83] Wang J. Wen W. Hanif M. Xia X. Wang H. Liu S. (2016). TaELF3-1DL, a homolog of ELF3, is associated with heading date in bread wheat. *Mol. Breed.* 36:161. 10.1007/s11032-016-0585-5

[B84] Weller J. L. Liew L. C. Hecht V. F. G. Rajandran V. Laurie R. E. Ridge S. (2012). A conserved molecular basis for photoperiod adaptation in two temperate legumes. *Proc. Natl. Acad. Sci. U.S.A.* 109 21158–21163. 10.1073/pnas.1207943110 23213200 PMC3529011

[B85] Wilhelm E. P. Turner A. S. Laurie D. A. (2009). Photoperiod insensitive Ppd-A1a mutations in tetraploid wheat (*Triticum durum Desf*.). *Theor. Appl. Genet.* 118 285–294. 10.1007/s00122-008-0898-9 18839130

[B86] Willige B. C. Zander M. Yoo C. Y. Phan A. Garza R. M. Trigg S. A. (2021). Phytochrome-interacting factors trigger environmentally responsive chromatin dynamics in plants. *Nat. Genet.* 53 955–961. 10.1038/s41588-021-00882-3 34140685 PMC9169284

[B87] Wilson P. Streich J. Borevitz J. (2015). “Genetics and genomics of *Brachypodium*,” in *Plant Genetics and Genomics: Crops and Models*, ed. Vogel J. P. (New York, NY: Springer International Publishing), 107–127. 10.1007/7397_2015_18

[B88] Woods D. Dong Y. Bouche F. Bednarek R. Rowe M. Ream T. (2019). A florigen paralog is required for short-day vernalization in a pooid grass. *Elife* 8:e42153. 10.7554/elife.42153 30618375 PMC6324881

[B89] Woods D. P. Amasino R. M. (2015). “Genetics and genomics of *Brachypodium*,” in *Plant Genetics and Genomics: Crops and Models*, ed. Vogel J. P. (New York, NY: Springer International Publishing), 259–273. 10.1007/7397_2015_10

[B90] Woods D. P. Bednarek R. Bouché F. Gordon S. P. Vogel J. P. Garvin D. F. (2017). Genetic architecture of flowering-time variation in *Brachypodium distachyon*. *Plant Physiol.* 173 269–279. 10.1104/pp.16.01178 27742753 PMC5210718

[B91] Woods D. P. McKeown M. A. Dong Y. Preston J. C. Amasino R. M. (2016). Evolution of VRN2/Ghd7-like genes in vernalization-mediated repression of grass flowering. *Plant Physiol.* 170 2124–2135. 10.1104/pp.15.01279 26848096 PMC4825116

[B92] Woods D. P. Ream T. S. Minevich G. Hobert O. Amasino R. M. (2014). Phytochrome C is an essential light receptor for photoperiodic flowering in the temperate grass, *Brachypodium distachyon*. *Genetics* 198 397–408. 10.1534/genetics.114.166785 25023399 PMC4174950

[B93] Yang Y. Peng Q. Chen G.-X. Li X.-H. Wu C.-Y. (2013). OsELF3 is involved in circadian clock regulation for promoting flowering under long-day conditions in rice. *Mol. Plant* 6 202–215. 10.1093/mp/sss062 22888152

[B94] Zagotta M. T. Hicks K. A. Jacobs C. I. Young J. C. Hangarter R. P. Meeks-Wagner D. R. (1996). The Arabidopsis ELF3 gene regulates vegetative photomorphogenesis and the photoperiodic induction of flowering. *Plant J.* 10 691–702. 10.1046/j.1365-313x.1996.10040691.x 8893545

[B95] Zakhrabekova S. Gough S. P. Braumann I. Müller A. H. Lundqvist J. Ahmann K. (2012). Induced mutations in circadian clock regulator Mat-a facilitated short-season adaptation and range extension in cultivated barley. *Proc. Natl. Acad. Sci. U.S.A.* 109 4326–4331. 10.1073/pnas.1113009109 22371569 PMC3306670

[B96] Zhao J. Huang X. Ouyang X. Chen W. Du A. Zhu L. (2012). *OsELF3-1*, an ortholog of *Arabidopsis EARLY FLOWERING 3*, regulates rice circadian rhythm and photoperiodic flowering. *PLoS One* 7:e43705. 10.1371/journal.pone.0043705 22912900 PMC3422346

