# Supplementary material for: EARLY FLOWERING 3 and Photoperiod Sensing in Brachypodium distachyon
Source: Front Plant Sci. 2022 Jan 6;12:769194. doi: 10.3389/fpls.2021.769194 (PMC8770904; doi:10.3389/fpls.2021.769194)
Supplement: Supplementary file 1 [file Table_1.pdf]

Table S1. Primers used in this study.

Purpose	Gene name	Gene ID	Primer	Sequence	Amplicon size	Reference
RT-qPCR	BdCCA1 (=BdLHY)	Bradi3g16515	FOR<br>REV	AGCTTGGCAGCGCATAGAAGAG<br>AGCATGGCTTCTGATTTGCACAG	64	Woods et al., 2014
	BdGI	Bradi2g05226	FOR<br>REV	GCATCTCCCGAAATGCAGATGTC<br>CACAAAGCGCATCTACAACCTTTC	79	Woods et al., 2014
	BdPPD1	Bradi1g16490	FOR<br>REV	CGATAATGGCAGTGGCACCCAAAG<br>TGTGGGCTGTCAATCTCCACAC	63	Woods et al., 2014
	BdVRN1	Bradi1g08340	FOR<br>REV	GTCGCGCTCATCATCTTCTC<br>TGCATAGGAGTAGCGCTCATAG	102	Schwartz et al., 2010
	BdVRN2	Bradi3g10010	FOR<br>REV	ATGCATGAGAGAGAGGCGAAGG<br>TCGTAGCGGATCTGCTTCTCGTAG	80	Ream et al., 2014
	BdFT1	Bradi1g48830	FOR<br>REV	TTCGGGAACAGGAACGTGTCCAAC<br>AGCATCTGGGTCTACCATCACGAG	129	Ream et al., 2014
	BdFTL9	Bradi2g49795	FOR<br>REV	GACAACTGGAGCTAGCTTTGGC<br>TGCGATGGATGCCGATTCTTGG	74	Woods et al., 2019
	BdUBC18	Bradi4g00660	FOR<br>REV	GTCACCCGCAATGACTGTAAGTTC<br>TTGTCTTGCGGACGTTGCTTTG	87	Ream et al., 2014
	BdACT3	Bradi4g41850	FOR<br>REV	CCTGAAGTCCTTTTCCAGC<br>AGGGCAGTGATCTCCTTGC	121	Li et al., 2011
Genotyping	Genomic ELF3	Bradi2g14290	FOR<br>REV	GGTGGTTTCAGCTTCTGCAG**G**TGAA<br>TCGCTGTCCCTAGATGCGTGGAATC	WT sequence cut by hpy166 but not the elf3 mutant	This article
	UBI:ELF3	Bradi2g14290	FOR<br>REV	CACCCATGCCTCCAATGTACTTCCC<br>ATCATGCGATCATAGGCGTC	615	This article

References

Li W (2011) Cloning and functional analysis of VRT2-like Genes in Brachypodium distachyon L. Tai'an, China: Shandong Agricultural University. 76 p.

Schwartz, C. J., Doyle, M. R., Manzaneda, A. J., Rey, P. J., Mitchell-Olds, T., and Amasino, R. M. (2010). Natural Variation of Flowering Time and Vernalization Responsiveness in Brachypodium distachyon. Bioenerg Res 3, 38–46. doi:10.1007/s12155-009-9069-3.

Ream, T. S., Woods, D. P., Schwartz, C. J., Sanabria, C. P., Mahoy, J. A., Walters, E. M., et al. (2014). Interaction of Photoperiod and Vernalization Determines Flowering Time of Brachypodium distachyon. Plant Physiol 164, 694–709. doi:10.1104/pp.113.232678.

Woods, D. P., Ream, T. S., Minevich, G., Hobert, O., and Amasino, R. M. (2014). PHYTOCHROME C Is an Essential Light Receptor for Photoperiodic Flowering in the Temperate Grass, Brachypodium distachyon. Genetics 198, genetics.114.166785. doi:10.1534/genetics.114.166785.

Woods, D., Dong, Y., Bouche, F., Bednarek, R., Rowe, M., Ream, T., et al. (2019). A florigen paralog is required for short-day vernalization in a pooid grass. Elife 8, e42153. doi:10.7554/elife.42153.
