# Supplementary figures and images for: EARLY FLOWERING 3 and Photoperiod Sensing in Brachypodium distachyon
Source: Front Plant Sci. 2022 Jan 6;12:769194. doi: 10.3389/fpls.2021.769194 (PMC8770904; doi:10.3389/fpls.2021.769194)

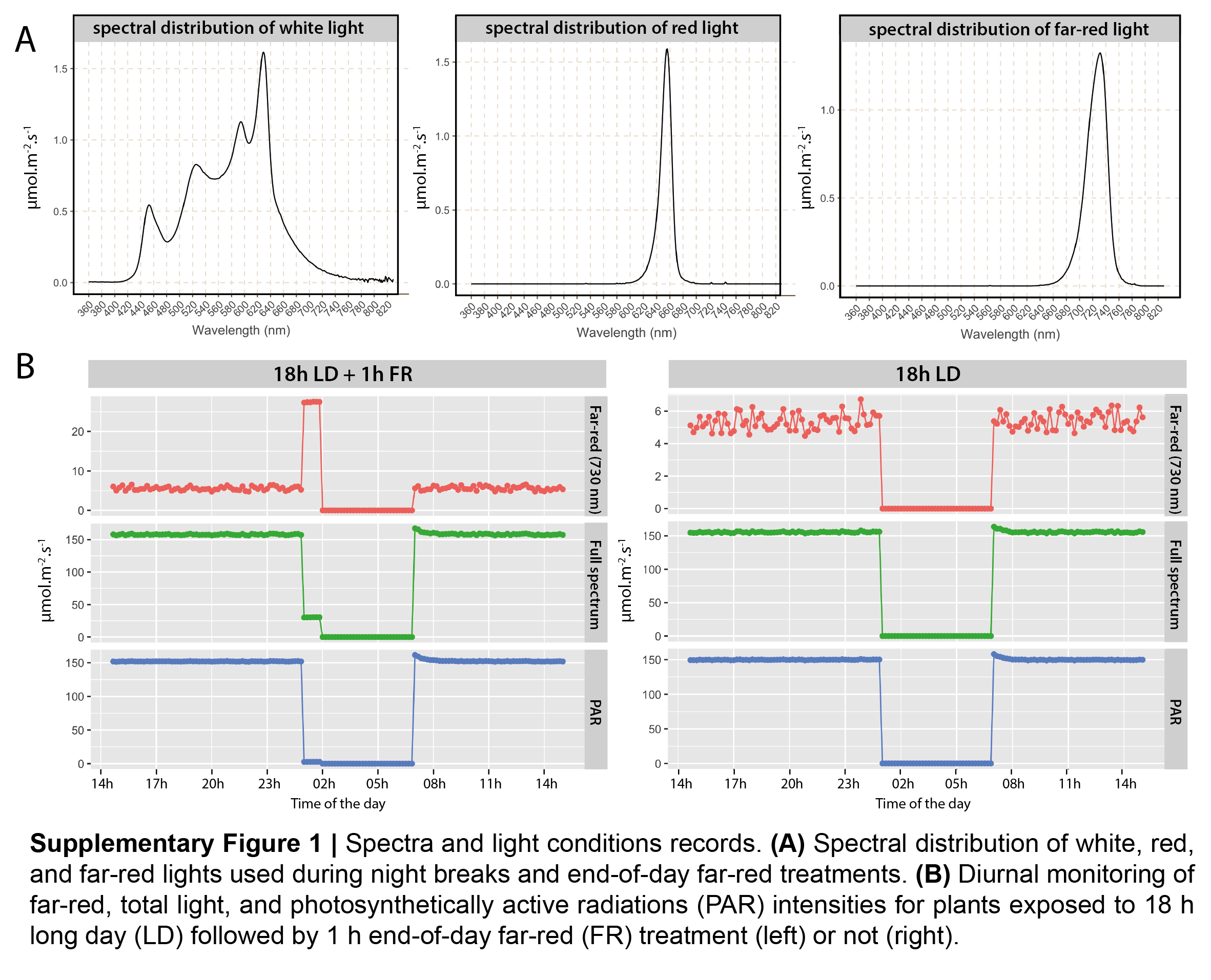

Supplement: Supplementary Figure 1 — Spectra and light conditions records. (A) Spectral distribution of white, red, and far-red lights used during night breaks and end-of-day far-red treatments. (B) Diurnal monitoring of far-red, total light, and photosynthetically active radiations (PAR) intensities for plants exposed to 18 h long day (LD) followed by 1 h end-of-day far-red (FR) treatment (left) or not (right). [file Image_1.png]

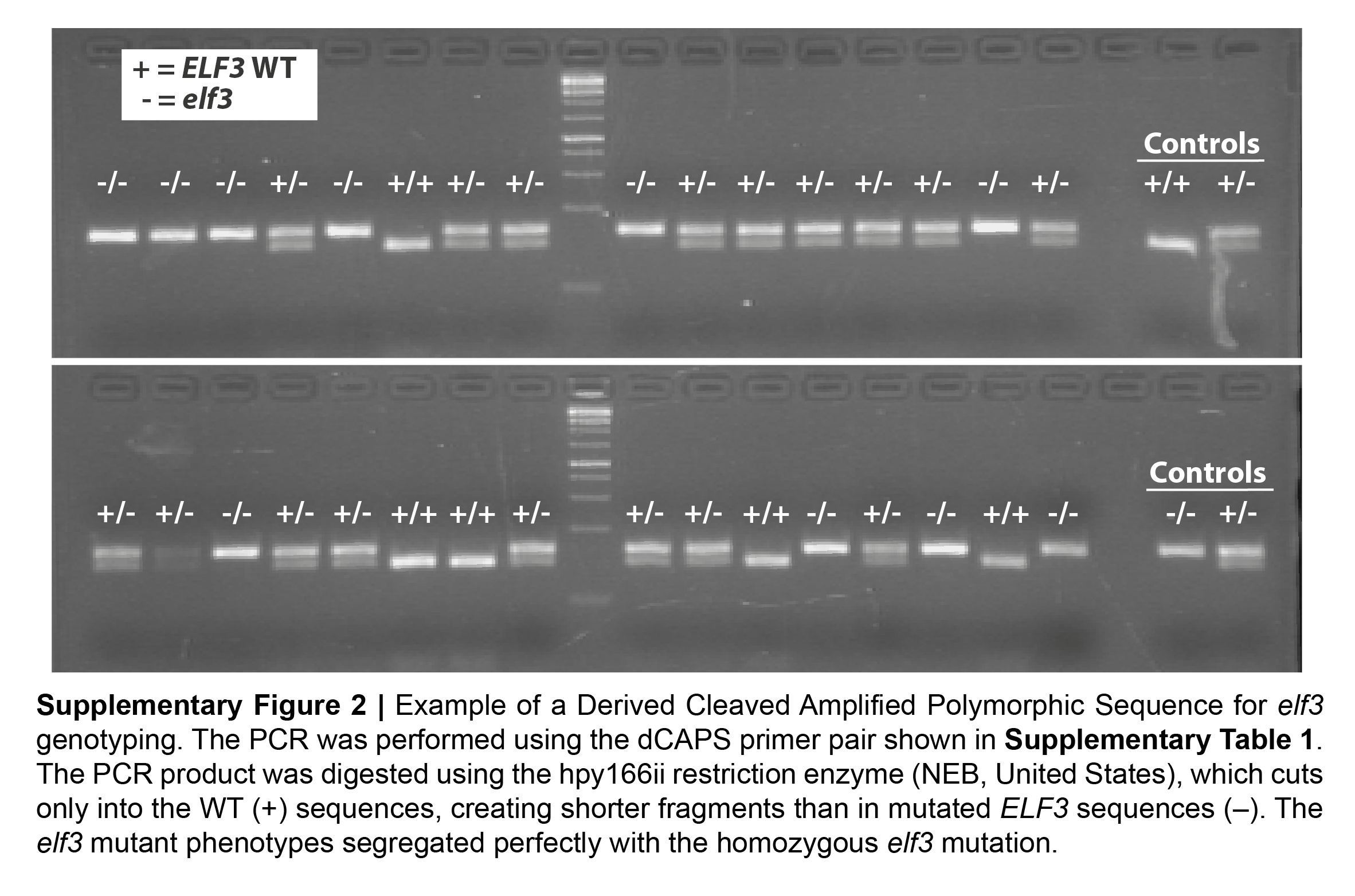

Supplement: Supplementary Figure 2 — Example of a Derived Cleaved Amplified Polymorphic Sequence for elf3 genotyping. The PCR was performed using the dCAPS primer pair shown in Supplementary Table 1. The PCR product was digested using the hpy166ii restriction enzyme (NEB, United States), which cuts only into the WT (+) sequences, creating shorter fragments than in mutated ELF3 sequences (–). The elf3 mutant phenotypes segregated perfectly with the homozygous elf3 mutation. [file Image_2.png]

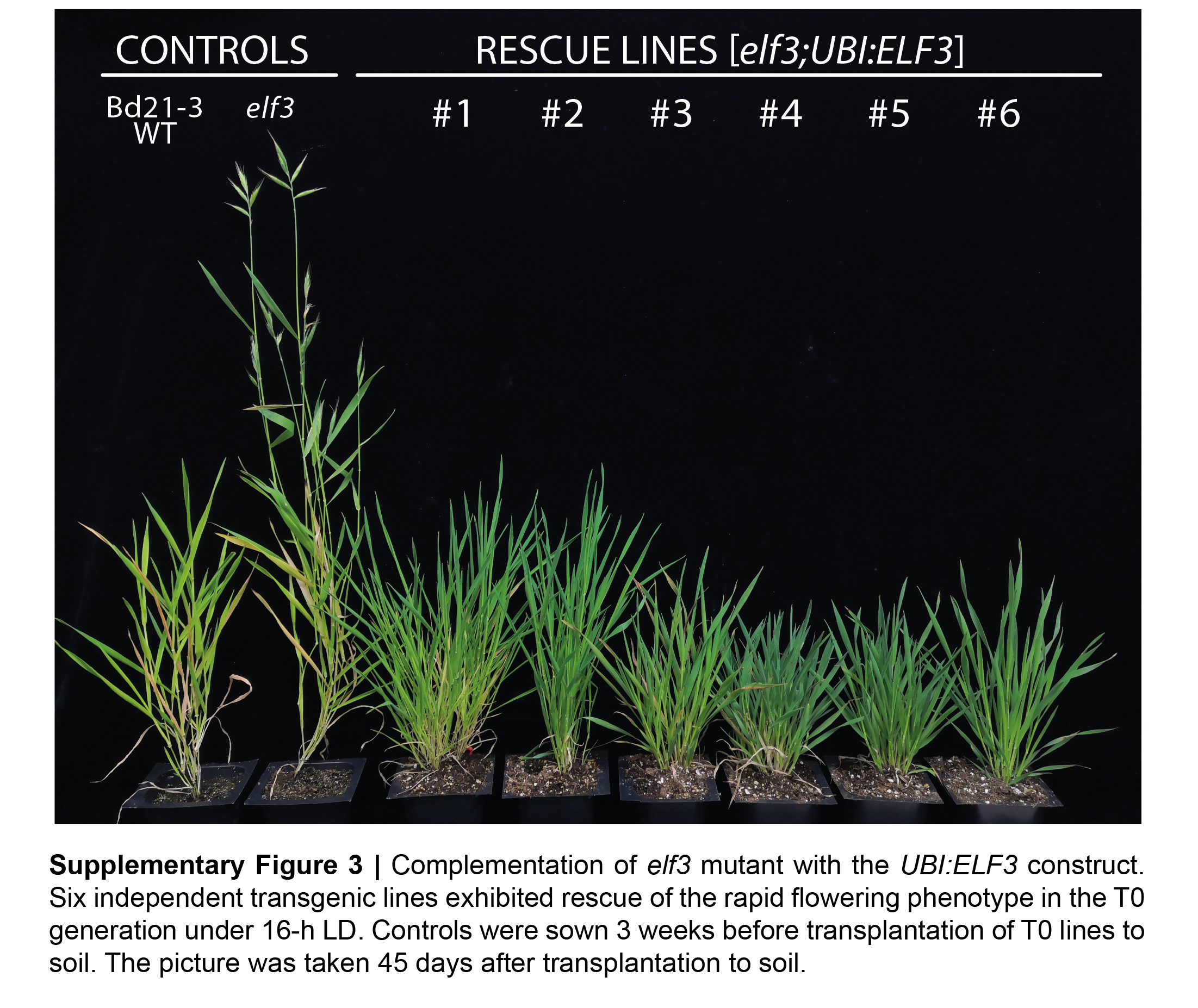

Supplement: Supplementary Figure 3 — Complementation of elf3 mutant with the UBI:ELF3 construct. Six independent transgenic lines exhibited rescue of the rapid flowering phenotype in the T0 generation under 16-h LD. Controls were sown 3 weeks before transplantation of T0 lines to soil. The picture was taken 45 days after transplantation to soil. [file Image_3.png]

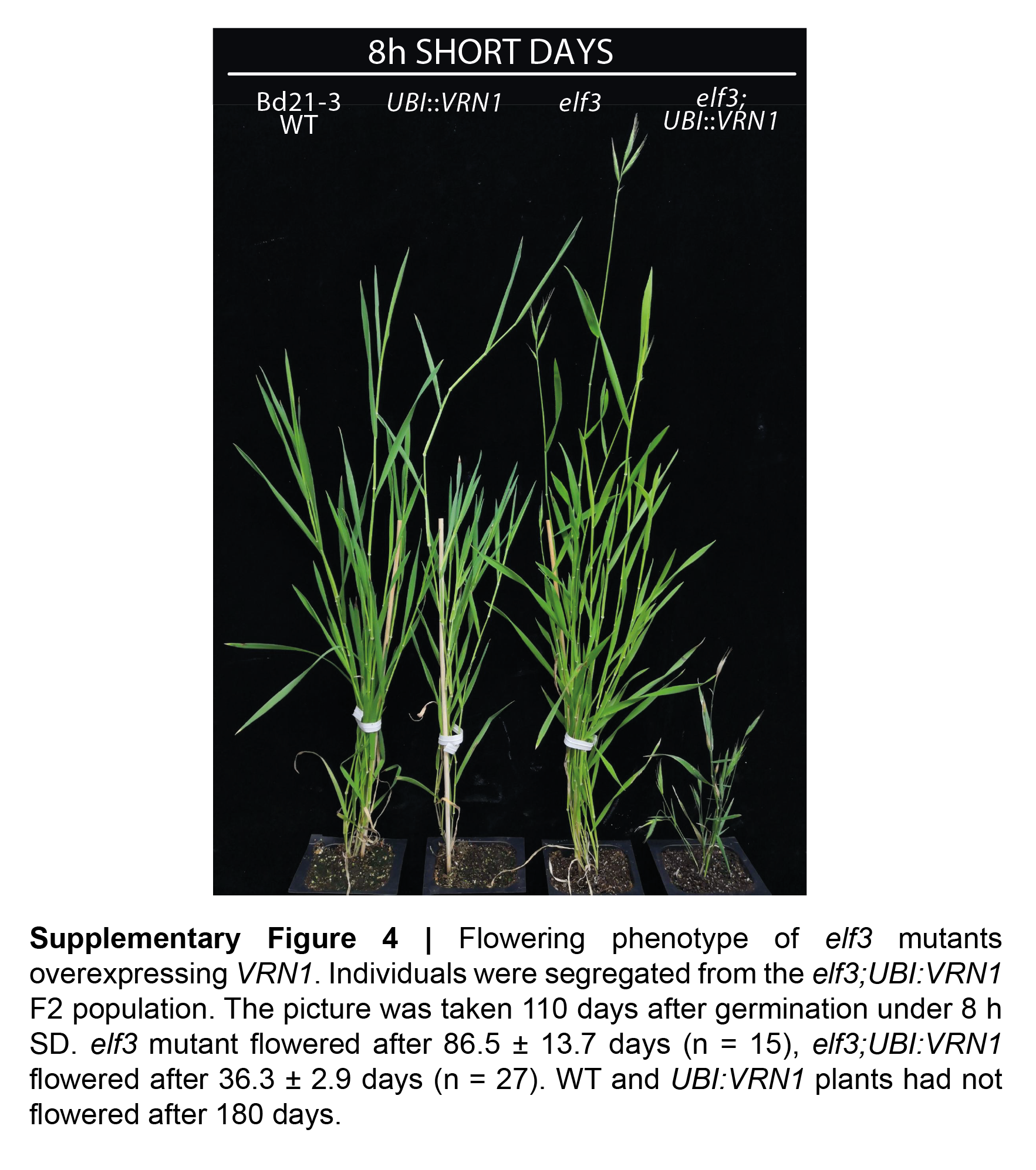

Supplement: Supplementary Figure 4 — Flowering phenotype of elf3 mutants overexpressing VRN1. Individuals were segregated from the elf3;UBI:VRN1 F2 population. The picture was taken 110 days after germination under 8 h SD. elf3 mutant flowered after 86.5 ± 13.7 days (n = 15), elf3;UBI:VRN1 flowered after 36.3 ± 2.9 days (n = 27). WT and UBI:VRN1 plants had not flowered after 180 days. [file Image_4.png]

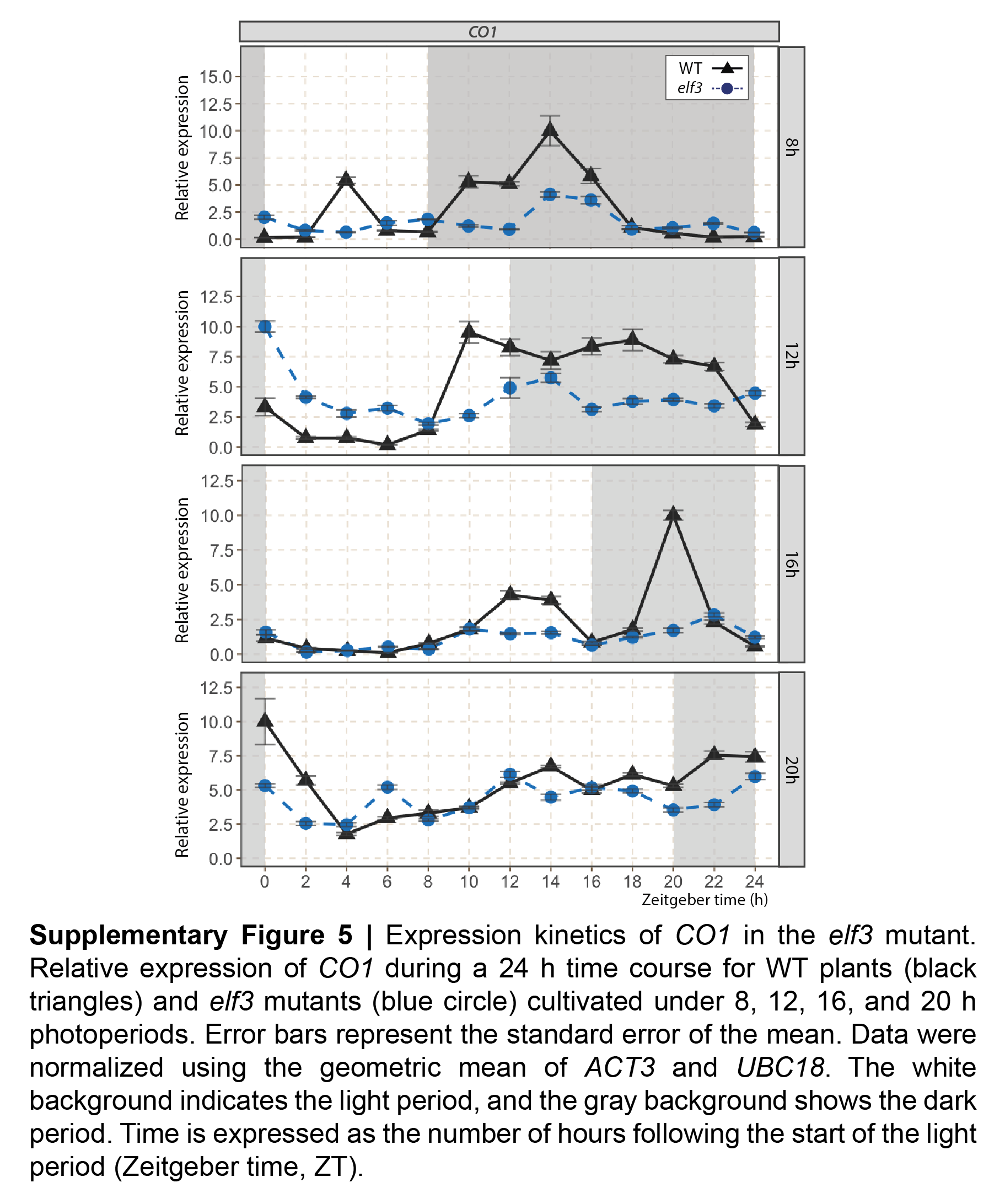

Supplement: Supplementary Figure 5 — Expression kinetics of CO1 in the elf3 mutant. Relative expression of CO1 during a 24 h time course for WT plants (black triangles) and elf3 mutants (blue circle) cultivated under 8, 12, 16, and 20 h photoperiods. Error bars represent the standard error of the mean. Data were normalized using the geometric mean of ACT3 and UBC18. The white background indicates the light period, and the gray background shows the dark period. Time is expressed as the number of hours following the start of the light period (Zeitgeber time, ZT). [file Image_5.png]
